# Mapping validity and validation in modelling for interdisciplinary research

**DOI:** 10.1007/s11135-020-01073-8

**Published:** 2020-11-20

**Authors:** Guus ten Broeke, Hilde Tobi

**Affiliations:** Biometris, Wageningen University & Research, Droevendaalsesteeg 1, 6708 PB Wageningen, The Netherlands

**Keywords:** Agent-based model, System dynamics, Interdisciplinarity, Measurement validity, Internal validity, Generalizability

## Abstract

Complex Adaptive Systems (CAS) is an interdisciplinary and dynamic modelling approach for the study of today’s global challenges. It is used for the explanation, description, and prediction of behaviours of system components and the system at large. To understand and assess the quality of research in which CAS models are designed and used, a thorough understanding of the meanings of ‘validity’ from social science research methodology and ‘validation’ from simulation modelling is needed. In this paper, we first describe the modelling process. Then, we analyse the concepts ‘validity’ and ‘validation’ as used in a set of research methodology textbooks and a set of modelling textbooks. We present one single map that integrates validity as characteristic of the model input, the modelling process, model validation, and the validity of the model built. The map is illustrated by means of one example. The terminology proposed in the map allows to describe and distinguish between the validity of primary research used for input in the model, how the quality of the modelling depends on structural and behavioural validation, and, how the assessment of the validity of the model is informed by these types of validation plus research with independent data.

## Introduction

Many of today’s global issues such as climate change, food security and social injustice, call for joint input of researchers from different scientific disciplines. The complexity of these issues has promoted the development of interdisciplinary research and complexity approaches, or as Fattore and Grassi (2015, p. 1551) put it “complexity is interdisciplinarity”.

Interdisciplinary research is (Aboelela et al. 2007, p. 341): “any study or group of studies undertaken by scholars from two or more distinct scientific disciplines. The research is based upon a conceptual model that links or integrates theoretical frameworks from those disciplines, uses study design and methodology that is not limited to any one field, and requires the use of perspectives and skills of the involved disciplines throughout multiple phases of the research process”. To make explicit what constitute distinct scientific disciplines, we use Kagan’s (2009) distinction of three distinct scientific cultures (2009): natural sciences, social sciences and humanities. In the Complex Adaptive Systems (CAS) context, the label ‘interdisciplinary’ refers to a team of people from at least the natural and the social sciences. We leave open whether the team includes researchers from the humanities.

One of the complexity approaches is modelling of CAS, which are composed of decision makers and their environment. Decision makers have goals, can interact, learn, and adapt within the environment. The system-level behaviour of a CAS emerges from interactions amongst decision makers and between individual decision makers and the environment. Examples of CAS are energy markets (North et al. 2002, with businesses, and electricity consumers as decision makers, within an environment composed of the power market and regulating administration), or land-use systems (Termansen et al. 2019, with farmers and land managers as decision makers, and the land and its vegetation as environment).

Keeping track of individual decision makers, their social interactions, their interactions with their physical environment and interactions between the social and physical aspects of the complex system is typically not feasible. Consequently, modelling is not only an important tool in the study of complex systems at the system level, but also for the social sciences that study social actors and support decision makers. Modelling a CAS may serve many purposes but three are unambiguously related to an empirical reality and will be considered in this paper: explanation, description, and prediction of new (i.e. not yet known) behaviours (Edmonds et al. 2019). For each of these purposes the modelling of a CAS builds on, and contributes to, different disciplines including the social sciences.

Hox (2017) suggested that social science research methodology could contribute to computational social sciences by exploring different kinds of validity. Because of the social sciences’ potential for the study of CAS, and vice versa, we take this suggestion to interdisciplinary computational sciences as represented by CAS modelling. After all, validity is an important quality criterion for all research and validation is an established criterion in the assessment of models. Since CAS modelling relies on input from the social sciences, validity of this input is important for the modelling process and conclusions. Because a common understanding and terminology presents one of the major challenges in interdisciplinary research (Fischer et al. 2011), the question rises how different definitions and uses of the concepts validity and validation relate to one another, and what a common terminology suitable for interdisciplinary research using CAS modelling, could look like. A common terminology would reduce confusion and facilitate debate about validity and validation of CAS models, but would also be useful in other interdisciplinary modelling approaches.

In the next section we describe the modelling process and adapt it to explicitly address the interdisciplinary nature of CAS. In the Methods section, we describe how we reviewed the use of the words validity and validation in research methodology and modelling textbooks, and how we synthesized both concepts with the adapted modelling cycle into the ‘map of validity and validation in CAS modelling’. The fourth section contains the results of this narrative review. In section five we present the synthesis: the map of validity and validation in CAS modelling. We use a CAS paper from the literature to show the full map from the validity of research results used as input in the modelling process, through model validation to the validity of the built model in section six. We conclude with a discussion of our work and suggestions to further explore and exploit the value of social sciences research methodology in the study of CAS.

## Interdisciplinary modelling of CAS

Five iterative steps are commonly distinguished in the modelling process: the first three go from reality to the conceptual model (the conceptual step), from the conceptual model to model code (the programming step), and, from the code back to the conceptual model (the code verification step). The fourth step is running the model, the fifth step the model validation (Augusiak et al. 2014).

The Methodology for Interdisciplinary Research (MIR) framework (Tobi and Kampen 2018) distinguishes four phases. The first is the conceptual design in which the what and why of the modules or sub-studies and the theoretical framework are decided on; followed by the technical design in which the how of the research is explicated. The operationalization of concepts connects the conceptual and technical phase as the theoretical framework chosen in the conceptual design informs decisions on measurements in the technical design. The data is collected and analysed in the execution phase, and, finally, in the integration phase the different modules of the interdisciplinary projects are appropriately combined and reported.

**Fig. 1:**
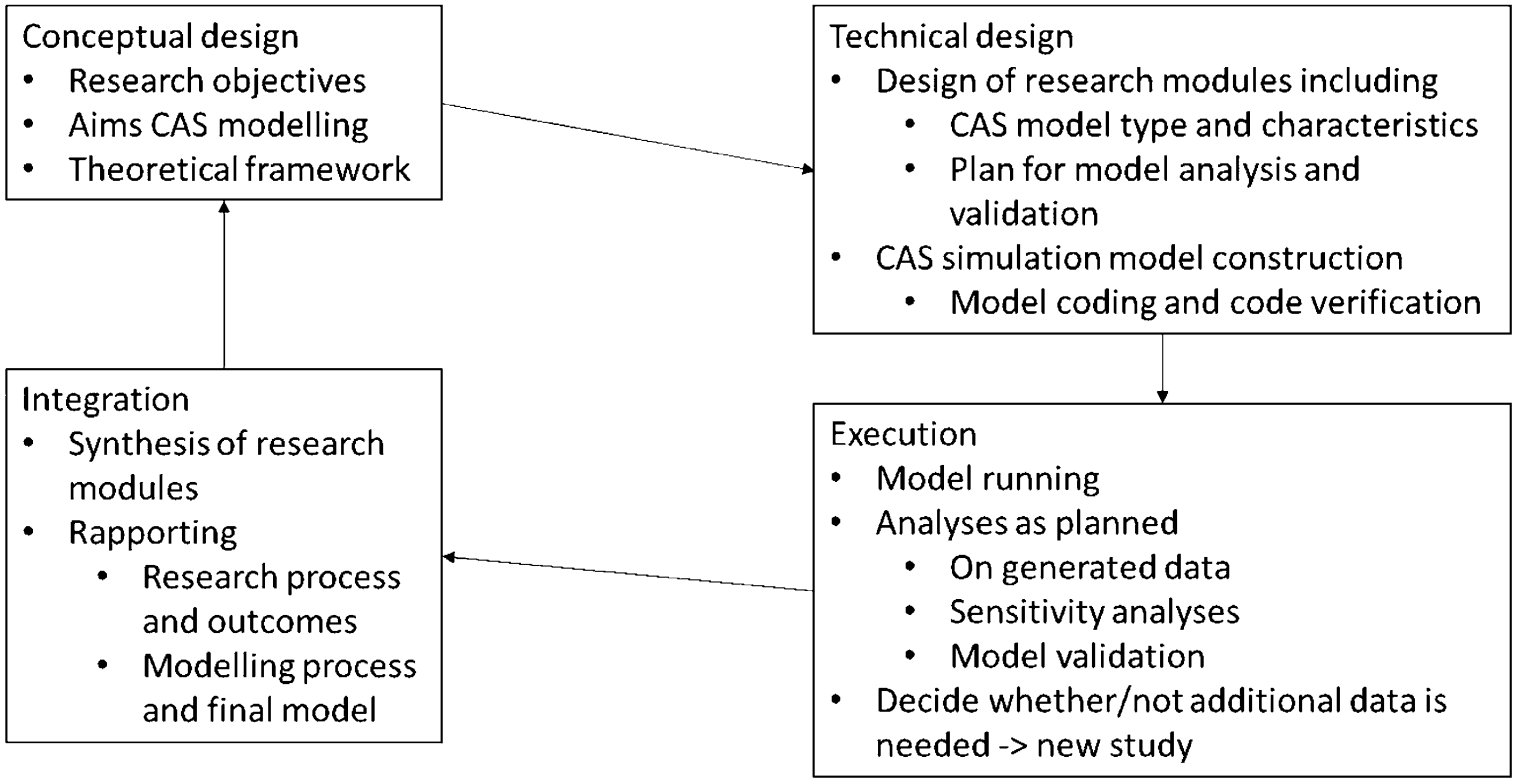
Model development for interdisciplinary research cycle

Combining the five modelling steps and the MIR framework into the adapted modelling cycle, we distinguish four different phases (see Fig. 1). In the first, the conceptual design phase, the interdisciplinary team members, together, decide on the research objectives and the aims of the CAS modelling. Theoretical frameworks are chosen to guide decisions on the system’s boundaries, components, relations and their operationalizations. This usually includes social as well as ecological or technological components.

The technical design (phase 2) consists of two distinct parts. The first one is the joint design of the different distinguished modules of the study (Tobi and Kampen 2018). For the modelling module this includes the type of model, how the components and relations distinguished during the conceptual phase are included in the model, how different parameter values will be chosen (e.g., by direct measurements by members of the interdisciplinary team, from literature or experts, or through calibration Hill 1998), and how the model and the data it generates will be analysed and validated. The second part of the technical design, the CAS model construction, is the coding of the model and the code verification. In this phase, the modellers may consult with the other team members when faced with issues not anticipated that call upon content matter knowledge.

The execution (phase 3) generally starts with running the model and doing the planned analyses. In sensitivity analysis the effects of changes and uncertainties in model parameters on the model output are investigated (Iooss and Lemaître 2015). Depending on the modelling aims, sensitivity analysis can test under what (if any) parameter settings the model generates the behaviour that it was aimed to explain, or it can reveal ranges of model predictions and their likelihood. If the aim is description, sensitivity analysis is less relevant (Edmonds et al. 2019).

The role of validation depends on the modelling aims too. When the aim is explanation, validation investigates whether the model presents an adequate explanation for the behaviour to be explained (Grimm and Railsback 2012). If the aim is description, validation will assess whether the model adequately represents all relevant elements to be described (Edmonds et al. 2019). For prediction, validation will be aimed at investigating whether model predictions match reality by comparing them against new data (Guerini and Moneta 2017). Concluding, validation yields information on what the model tells about the system, related to the aims of the research. Consequently, the interpretation of this validation is always an interdisciplinary team effort.

The fourth phase (integration) involves, where appropriate, a synthesis of different research modules, and reporting. Different publications may address different modules and although the leading authors may differ, all publications require interdisciplinary input.

Since the system-level behaviour of CAS originates from interactions within the system, studying the within-system processes is essential for theory building and understanding CASs. Consequently, we focus on the most commonly used modelling approaches for representing these processes: system dynamics models (Phelan 2015; Schieritz and Milling 2003) and agent-based models (ABM) (Filatova et al. 2013). System dynamics models describe feedbacks between system components on an aggregated level and simulate the resulting development of the system over time. For example, Hsiao et al. (2012) have studied the development of professional baseball in Taiwan by describing feedbacks between several variables, including the number of fans, team income, player quality, and team image. They used their model to predict the development of these variables over time. ABMs are composed of interacting agents (e.g., people, animals, companies, etc.), with individual characteristics and behavioural rules. For example, Holtz and Pahl-Wostl (2012) have developed an ABM to better understand how farmers’ characteristics influence land-use changes in Upper Guadiana, Spain. The farmers are modelled as agents that are influenced by social factors (e.g., policies, risk aversion, knowledge of crop-irrigation technology), ecological and technological factors (e.g., possible crops, irrigation technologies), and relations amongst and between those components (e.g., respecting water law, crop-irrigation combinations, profit).

## Methods

This section provides details on how the inventory of validity and validation terminology and its synthesis with the adapted modelling cycle were made (Fig. 1).

### Inventory of ‘validity’ and ‘validation’ in research methodology and modelling textbooks

The small convenience sample of textbooks (n = 11) was based on hands-on availability, while ensuring different focusses and takes on social research methodology (see Table 1). The textbooks’ indices and glossaries were hand-searched for references to validity and specific validities or validations. All mentioned keywords under ‘Validity’ and ‘Validation’ were collected. Each different keyword was made a column in an excel file and each book a row. Definitions and descriptions for each keyword were read, and extracted and entered in excel unless they were duplicates (within books) or the description was too implicit to interpret. Finally, to ensure completeness, the full index of each book was searched again for all separate keywords retrieved from any of the books. The excel file is available from the corresponding author upon request and “Appendix 1” contains a summarized version.

**Table 1:** Description of methodology and modelling textbooks included

Book number	Methodology textbooks		
	Reference (alphabetically ordered on first author)	Focus	Aimed Audience*
1	Bowling, A (3rd, 2009) Research methods in health. Maidenhead, UK: Open University Press, McGrawHill	Health and Medicine	Graduate students and researchers
2	Bryman, A (4th, 2012) Social research methods. Oxford: Oxford University Press	Sociology and politics	(Under)graduate students
3	Creswell, JW (4th, 2014) Research design: qualitative, quantitative, and mixed methods approaches. Los Angeles: Sage	Social sciences	(Under)graduate students, researchers
4	Deming, ME and Swaffield, S (2011) Landscape architecture research. Hoboken NJ: Wiley	Landscape architecture	Graduate students, researchers, and designers*
5	Freankel, JR and Wallen, NE (7th, 2009) How to design and evaluate research in education. Boston: McGraw-Hill	Education and learning sciences	(Under)graduate students*
6	Gray, DE (4th, 2018) Doing research in the real world. Los Angeles: Sage	Social Sciences	(Under)graduate students*
7	Kumar, R (5th, 2019) Research methodology; a step-by-step guide for beginners. Los Angeles: Sage	Social sciences, and health sciences	(Under)graduate students
	Modelling textbooks		
	Reference	Focus	Aimed Audience
8	Bala, BK et al. (2016) System dynamics: modelling and simulation. Singapore: Springer	System dynamics	(Under)graduate students, researchers, practicing system dynamists, policy planners
9	Duggan, J (2016) System dynamics modelling with R. Switzerland: Springer	System dynamics	(Under)graduate students, researchers, engineers, consultants
10	Gilbert, N (2008) Agent-based models. Thousand Oaks, CA: Sage	Agent-based modelling (social sciences)	(Under)graduate students and researchers
11	Railsback, S and Grimm, V (2nd, 2019) Agent-based and individual-based modeling. A practical introduction. Princeton, NJ: Princeton University Press	Agent-based modelling (ecology)	(Under)graduate students and researchers

^*^Aimed audience according to book’s author or, with*, as perceived by the authors of this paper

All entries in the Excel file were read by both authors independently. Then descriptions of each keyword were discussed for commonalities and differences within and across the sets of textbooks. The keywords and identified commonalities and differences are described in the results Sects. 4.1–4.3. In the remainder the books in Table 1 are referred to by ‘B’ followed by the number.

### Synthesis

For the synthesis, all identified keywords were compared across both groups of textbooks, and their relation with the adapted modelling cycle was discussed. Sketches of the position of keywords in a terminology network were made and discussed in a number of rounds until the categorisation of included keywords on the map was agreed on by both authors. Internal consistency of the map was checked by means of a model of a fishery system (Libre et al. 2015), which is presented as an illustration in Sect. 5.

## An inventory of keywords in textbooks

### Validity and validation in methodology textbooks

A total of 26 different keywords were found in the methodology textbooks (“Appendix 1”).

Most textbooks pay attention to the ability of an instrument to measure what it is designed to measure, although by different names, amongst others validity, internal validity, construct validity, and, measurement validity. Hypotheses about how values on the instrument under study ought or ought not correlate with other variables, are required to assess convergent (B1 and B2) and discriminant validity (B1) respectively. The degree to which the scores on an instrument predict future characteristics reflects its predictive validity (B1, B2, B5, B6, and B7). Face and content validation do not call upon statistics, nor on hypotheses about relations between measures. Kumar (p. 272) describes the process of face validation as linking the individual items in a research instrument with the aim of the research. Other textbooks provide similar descriptions (B1, B2, B6). Content validity relates to the extent the instrument covers the full scope of the concept it measures (B1, B6, B7). In summary, we opt for measurement validity as the label for the ability of an instrument to measure what it is designed to measure, which encompasses construct, convergent, discriminant, predictive, concurrent, face, and, content validity.

The textbooks used ‘internal validity’ differently between and within textbooks. We regard internal validity as an attribute of the research’s conclusions, relating to claims about (particularly causal) relations between variables (following B1, B2, B4, B5, and, B6). This implies that internal validity is determined by the quality of the study design.

Other keywords referring to the generalizability of research results were ecological validity, external validity, and, transferability. We will understand external validity as the ability to draw inferences from the sample to the population of interest (following B1, B5, and, B6), which implies that external validity is determined by the sampling design of the research. Ecological validity is understood as the generalizability of research findings to other populations and other, naturally occurring, settings (B1, B2 and B5). This implies that ecological validity is influenced by many aspects of research design. Transferability, used in the context of qualitative research, parallels generalizability as “the degree to which an individual can expect the results of a particular study to apply in a new situation or with new people” (Fraenkel, G-9). Concluding, generalizability consists of external and ecological validity.

Attributes such as trustworthiness (with components such as credibility, transferability, dependability and confirmability) and authenticity relate to particular qualitative research traditions in which the concept validity is considered inappropriate and replaced by other quality criteria (B2, B3, and, B6). Statistical validity was defined as the appropriateness of research methods used and the presence of sufficient statistical power (B3 and B6). In our view, trustworthiness and statistical validity fully overlap with the validities discussed above. The keyword validation referred to the evaluation of professional development programmes (B6).

In summary, this analysis yielded three general classes of validity: measurement validity relating to the data obtained, internal validity relating to claims on (causal) relationships, and generalizability of research results.

### Validity and validation in modelling textbooks

Out of the 43 identified keywords related to validity and validation, 18 were present in at least one of the modelling textbooks. Most keywords were consistent between both system dynamics textbooks, but the two ABM textbooks used different terminology. The only term that appeared in all four textbooks was sensitivity analysis, and the meaning was consistent between the four textbooks (B8–B11 in Table 1).

Both system dynamics textbooks (B8 and B9) distinguished consistently between behavioural validity, and structural validity. Behavioural validity refers to “how well the model output aligns with the observed real-world behaviour” (B9 p. 125). Structural validity refers to the model inputs, components and their relationships. Structural validity is based on “comparison with knowledge about the real-world system structure” (B9 p. 124). Several aspects of structural validity were distinguished in both system dynamics textbooks, each corresponding to different tests: boundary adequacy tests, dimensional consistency tests, extreme condition tests, structure confirmation tests, and parameter confirmation tests. Boundary adequacy tests assess whether all the relevant system components are represented in the model (B9 p. 125). Dimensional consistency tests verify the consistency of dimensions of model equations (B9 p. 125). Extreme condition tests investigate the plausibility of the model behaviour at extreme parameter conditions (B9 p. 125), whereas structure and parameter verification tests evaluate the model structure and parameter values against knowledge of the real-world system (B9 p. 125–6). For each of these tests, Bala et al. (p. 216) refer to verification tests rather than confirmation tests, but the meaning is the same as in Duggan (B9).

Duggan (B9) distinguished between validation for correlational models and for causal-descriptive models. Correlational models are black-box models that are solely aimed at predicting or reproducing data from the real-world system. Duggan (B9, p. 123) considers behavioural validation to be important for black-box models, but structural validation to be irrelevant. Causal-descriptive models, in addition to predicting or reproducing data from the real-world system, aim to give a good representation of the underlying processes. Thus, for causal-descriptive models Duggan considered both behavioural and structural validation to be important.

The ABM textbooks (B10 and B11 in Table 1) offer a general definition of validation, but neither distinguished different types. Railsback and Grimm (B11, p. 244) defined validation as “the process of searching for and testing independent predictions”. This testing is done by comparing model output to new data from the real-world system. Gilbert (B10, p. 80) defined validation as “the process of checking that the model is a good representation of the target”. Again, this is done by comparing model output to observations of real-world systems. Thus, neither of the two ABM textbooks mentioned validation of model inputs, nor the structure of the model.

Gilbert (B10, p. 41–6) discussed how the process of validation depends on the level of abstractness of the model. Models of specific systems can generate specific predictions to be used in validation. In contrast, output of abstract models cannot be matched directly to a specific system.

### Comparison between research methodology and modelling textbooks

The methodology textbooks distinguished many different kinds of validity, with little consensus. Threats to different kinds of validity could be found, only implicitly informing the process of validity assessment. In contrast, modelling textbooks mentioned validation as process only, without explicating the attribute being investigated by means of that process. The one keyword with a similar meaning in both methodology and modelling textbooks is “sensitivity analysis”. Sensitivity analysis appeared in one methodology textbook as “method of estimating the robustness of the conclusions of the study or its assumptions” (Bowling, p. 169).

Concluding, taking Hox’s suggestion (2017) to interdisciplinary computational modelling: social science research methodology may contribute to interdisciplinary modelling by making the attributes investigated by means of validation explicit. In turn, interdisciplinary computational sciences may remind social science research methodology to distinguish between the attribute, its threats and its assessment.

## Synthesis

**Fig. 2:**
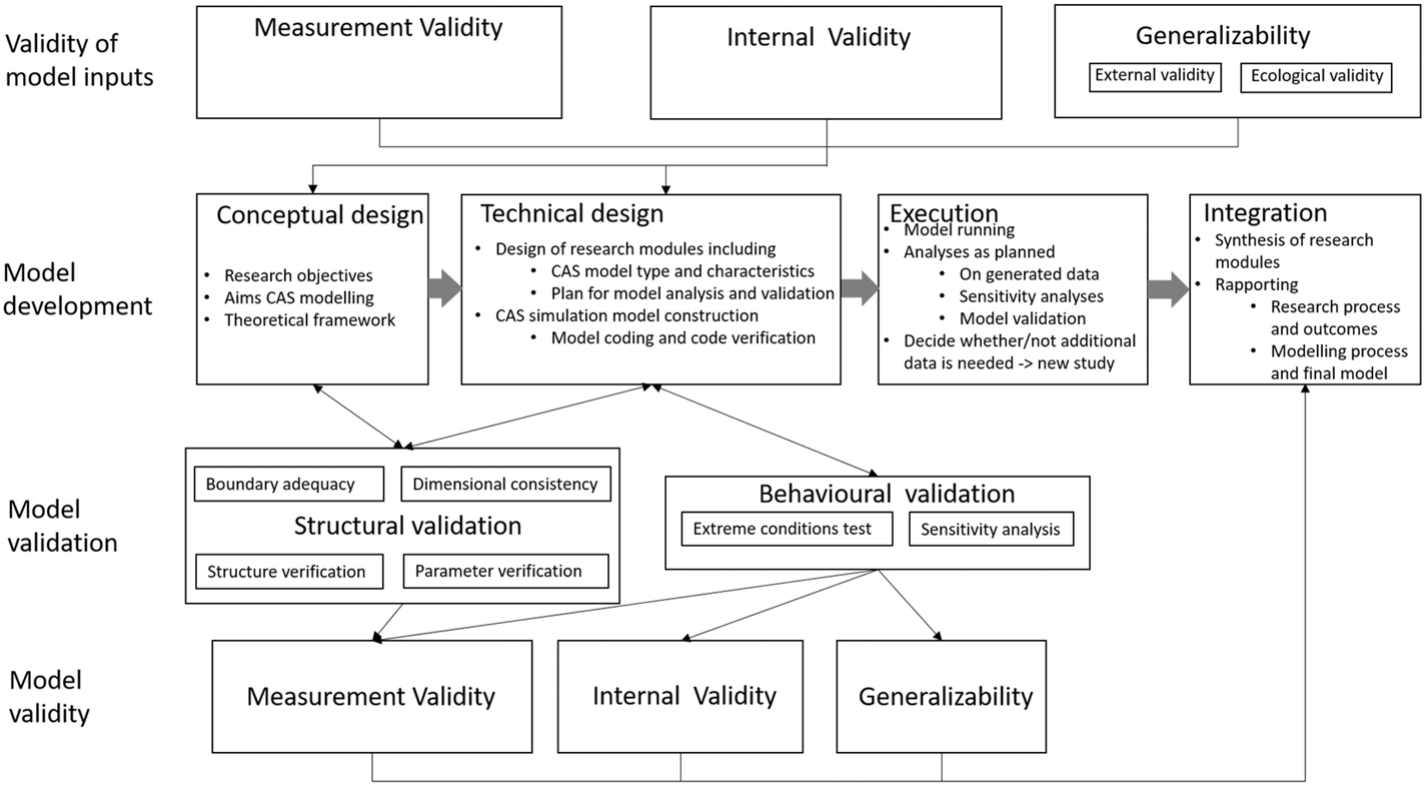
Map of validity and validation in CAS modelling

In this synthesis we present the `map of validity and validation in CAS modelling’ (Fig. 2). Validity as we have seen it in the research methodology books, is a quality characteristic of the model inputs. This forms the first layer in the map. The second layer depicts the modelling cycle (Fig. 1). The third layer “model validation” depicts model validation processes, and is based on the review of validation in the modelling textbooks. The bottom layer is about model validity. In the following three sections, we focus on how the layers inform one another to conclude with three classes of validity of models for interdisciplinary research.

### From validity to model development

The three classes of validity of primary studies inform model development in different ways (see Fig. 2). The measurement validity of primary studies informs the theoretical framework, f.e. based on (lack of) measurement validity the interdisciplinary team may prefer one concept over another one (e.g. prefer ‘sex’ over ‘gender’). Measurement validity also informs the choice of meaningful default parameter values in the technical design and parameter ranges in sensitivity analyses.

In general, internally valid studies are needed to inform the theoretical framework, the kind of relationships (e.g. causal, confounded, mediated or moderated) in the model design, and the design of sensitivity analyses. Generalizability of primary studies determines whether results obtained in a different setting or in a small selection of system components may be considered informative for building the theoretical framework and the model.

### Towards model validation

Structural validation assesses the design of the model. Each of the four tests in Fig. 2 is aimed directly at assessing a specific element of this design, as described in Sect. 4.2. For example, any errors revealed by a dimensional consistency test point towards errors in equations of the model.

Execution of the behavioural validation is part of the execution phase, and the outcomes of this validation also relate to the technical design phase. Extreme conditions tests may reveal problems in the technical design related to either the conceptual model, or the coding. Sensitivity analysis helps assess choices made in the technical design by verifying to what extent different components of the model influence the model output and resulting conclusions. Furthermore, the outcomes of sensitivity analysis will reveal whether the sensitivity analyses planned in the technical design were sufficient in relation to the aims of the modellers, or whether additional analysis is needed.

The outcomes of structural and behavioural validations rely on the quality of the conceptual and the technical design, and may lead to changes in the conceptual design, the technical design and the analyses planned, hence the double-headed arrow between the second and third layer in Fig. 2.

### From model validation to model validity

Finally, the results of model validation inform on the validity of the model. Here, we distinguish between measurement validity, internal validity and generalizability.

Measurement validity of models is about whether the model yields data that represent the real-world system in a way suitable for the research question. This is influenced by the quality of the primary studies, the theoretical framework and the quality of the coding. It is furthermore determined by the rigorousness of both the structural and behavioural validation; and how the researchers use the validation procedures’ outcomes. The assessment of the measurement validity of the model can take on many different forms, including expert judgement.

The internal validity of the model is about the quality of the conclusions on relationships between and across system components over time, as drawn from the model. The internal validity of the model is limited by the quality of the primary studies, the theoretical framework, and the quality of coding. It may be enhanced by information obtained through behavioural validation, in particular sensitivity analysis. The internal validity of a model may be assessed through the collection of new data obtained in the real-world or in an experimental study design.

Generalizability of the model relates to the usefulness of the model in different settings (time, place, or research question) than the one it was designed for. This is determined not only by the choosing the right primary studies and the theoretical framework, but also by tests of the robustness of the model results and conclusions (i.e. sensitivity analysis). As behavioural validation does not relate a sample to a population, nor a particular setting with other real-life settings, we chose not to distinguish external and ecological validity.

Two common purposes of interdisciplinary modelling studies are (1) gaining a better understanding of the system under study by means of description or explanation, and (2) using the model for informing policy by means of prediction. For the first aim, it is essential that the model is a good representation of the real-world system, that is that the model has measurement and internal validity. Assessment of the model’s validity should therefore focus on how the system-level behaviour of the model is explained by underlying processes. For the second aim, informing policy, additional focus is needed on the predictive validity of the model, and the generalizability of the model to different settings.

## Illustration: the case of the Philippine tuna purse seine fishery

Our aim with this illustration is to show how Fig. 2 facilitates discussion and assessment of validation and validity of CAS models. We selected a study on Philippine fishery system (Libre et al. 2015) for several reasons. First, the study has a clear interdisciplinary character, using inputs from biology, economics, and sociology. Second, the study describes the entire process of model development, including preliminary data gathering. Thirdly, the study used primary and secondary data from various sources as model inputs, which makes it a suitable example of what this input data implies for the validation and validity of the model. Table 2 summarises all the steps in the process of data collection and model development, as described by Libre et al. (2015). No additional data was gathered, beyond what was reported in Libre et al. (2015). In the following subsections, we use the map of Fig. 2 to discuss what the description of Libre et al. (2015) implies for the model validation and validity. Each of the following subsections corresponds to one layer in Fig. 2.

**Table 2:** Modelling steps in Libre et al. (2015)

Modelling step	Description
Choice of model	An ABM was chosen, in order to represent decisions of individuals
Interviews	Interviews were conducted with fishers and other stakeholders, to gather data as input for the model
Additional data gathering	Reports and studies from BFAR and WCPFC served as further input data for the model
Conceptual model development	An ABM was developed based on gathered data, and theories taken from previous literature
Parameter setting	Setting of parameter values was based on interview data
Verification	Checking and testing of code was done independently by two co-authors, in an iterative process with the main coder
Validation	1000 runs at default parameter settings were compared against existing data on fish catch
Sensitivity analysis	A number of model parameters were varied over a wide range to assess their individual effect on model output

Libre et al (2015) recognized that models that inform policy and represent fishers as perfectly rational economic agents show several problems. Therefore, their aim is (p. 251) “to view fisheries as CAS to evaluate the effects of social factors and bounded rationality on macro-level outcomes of fisheries, including the number of vessels, total fishing days (the measure of fishing effort), fish stock, and industry profit”.

### Validity of model inputs

The authors relied on a number of primary studies, and also collected primary data themselves. In total, 44 interviews were conducted with various types of stakeholders. Measurement validity of the collected primary data was not mentioned by the authors, but they do provide information that allows some assessment. Face and content validity of model input that relied on the interviews may be assessed based on the interview guide (Libre et al. 2015, Appendix A). Like in any study of CAS, the authors had to deal with incomplete information on some aspects of the system, e.g. they lacked information on spatial distributions of catch, or stock size.

Assumptions about causal relationships between variables were drawn mostly from interview data. The internal validity thus depends on the insights of interviewed stakeholders, and is difficult to assess.

The interviews were conducted with Philippine tuna purse seine fishery stakeholders. The generalizability is difficult to judge without thorough background knowledge of the fishery and without knowing how interviewees were selected. The parametrisation of the model was based on all Philippine skipjack tuna catch and fishing efforts as registered at ports and collected by the Philippine Bureau for Fisheries and Aquatic Resources and by the Western and Central Pacific Fisheries Commission.

### Model development

The authors discussed clearly the aims, chosen theoretical framework, the choice of model type, and the further steps of model development, as summarised in Table 2.

### Model validation

Structural validation: the authors do not discuss structural validation but provide clear information about the model boundaries: the modelled entities, spatial and temporal scales, and state variables. However, the boundary adequacy also depends on the internal, external, and measurement validity of the interview data, since choices on what should be included in the model were informed by this data. So, since validity of the interview data was difficult to assess, the same holds true for the boundary adequacy. Any reader may perform dimensional consistency tests, since dimensions for each parameter (Table 4, p. 256) and all model equations (online appendix) are available. The reader may also perform parameter verification, as default values and their sources are provided, as well as the range of values used in the sensitivity analysis. Regarding structure verification, the authors justify most of the modelling choices based on the interview data. However, for the exact formulation of some model processes only little justification is provided by the authors.

Behavioural validation: the authors report a sensitivity analysis in which each investigated parameter was individually varied over its maximum range. This analysis is a combination of extreme condition testing and local sensitivity analysis (i.e., sensitivity analysis in which each parameter is varied individually). Parameters excluded from this analysis were listed as well (Libre et al. 2015, Appendix A). The sensitivity analysis was used to identify influential parameters, and to show that several social factors are relevant for the model outcomes. Detailed results are reported for several key parameters, whereas for the other parameters a summary is reported. No global sensitivity analysis (i.e., a sensitivity analysis in which all parameters are varied simultaneously) was performed.

### Model validity

The assessment of the validity of this model, requires new independent research, but also depends on the structural and behavioural validation. Here, we discuss these dependencies, and how further assessment of the validity of this model might be approached.

Measurement validity is informed by the structural and behavioural validation of the model. Structural validation should ensure that the variables included and produced by the model are relevant for the research question, and depends on the validity of the interview data, which could not be judged. Behavioural validation should ensure that relevant parameter settings are explored, which for our example has been done through sensitivity analysis. A potential way to assess face and content validity for the presented model is through independent expert judgment. Furthermore, model details could be presented to stakeholders and experts to assess whether the decision-making of fishers and fishing companies in the model is realistic. Predictive validity could be assessed by comparing model projections with new data on catch and industry profit.

Structural and behavioural validation are prerequisite to assess internal validity of the model. For the present case, the abovementioned dependency of the structural validation on the interview data affects the internal validity of the model. The reported behavioural validation, in the form of sensitivity analysis, revealed that some relations between agents are influential on the model outcomes. New data would need to be collected to verify whether these relations were modelled correctly. The authors were interested in the Philippine tuna fishery, and, consequently, included factors and parameter values specific to that fishery in the Philippines. Whether generalization to other fisheries in the region is meaningful, requires further research. The same holds for the use of the model in other economic sectors, or on other timescales. The importance of factors specific to the local situation in the Philippines, or to the economic sector, could be assessed using additional global sensitivity analysis. Such an analysis would yield insight into the generalizability of the model, but is not presented by the authors.

## Discussion

We identified three basic validities of primary studies from research methodology (measurement validity, internal validity, and generalizability) and two types of validation from computer modelling (structural validation and behavioural validation). Interdependencies between validities and validation of the conceptual model, the model inputs, the modelling process, and, the model as research instrument were identified and illustrated. Different aspects of validity and different processes for validation were disentangled, resulting in a map with four layers (Fig. 2) to facilitate communication in modelling for interdisciplinary research. Furthermore, we applied the map to an example to show how the map can help discuss and understand validity and validation in the modelling process and the model’s assessment more precisely. For this example, model validity was difficult to establish, partly because the validity of model inputs was difficult to establish and not only because additional research is needed. This illustrates that the map facilitates communication about validity, validation, and interdependencies in interdisciplinary CAS modelling.

In the scientific literature, validity and validation is discussed along disciplinary lines, like economics (Fagiolo et al. 2019; Windrum et al. 2007), land-use and planning (Brown et al. 2005), and, ecology (Augusiak et al. 2014). There appears to be no bridge, which confirms the importance of the present study. In economics, validation is discussed as the process of matching model output against real-world data, and is usually discussed alongside methods for calibration and sensitivity analysis (Fagiolo et al. 2019; Windrum et al. 2007). In land-use science, Brown et al. (2005) distinguished between validation of the modelled processes, and their final outcomes which is akin to what we call assessment of predictive validity and internal validity of the model. In ecology, Augusiak et al. (2014) proposed ‘evaludation’, as the merger of model validation and model evaluation, which became the foundation of the modelling documentation framework TRACE (Grimm et al. 2014; Schulze et al. 2017). We see the merits of ‘evaludation’ and the TRACE framework, but neither distinguishes between the validity of model input, validation of the model, and, model validity, like this paper does. Also within ecology, Getz et al. (2018) distinguished the modelling process from the model as a product, but did not go into details with respect to what is (cross-) validation of models and validity of models.

Interdisciplinary CAS models rely on inputs from various sources and the quality of a model inevitably depends on the quality of these inputs. The validity of interdisciplinary models is therefore vulnerable to their inputs regardless of the disciplinary backgrounds. This emphasizes the importance of validity as we know it from research methodology to all disciplines that aim to contribute to CAS models. If the quality of these models was to be assessed purely based on validation outcomes ignoring validity of inputs this could easily lead to inappropriate model structures, assumptions and outcomes. While validity of models will always rely on inputs, our proposed map (Fig. 2) makes this reliance explicit by showing how validity of model inputs impacts on different steps in the modelling process and, ultimately, on the validity of the resulting model. By explicating terminology, our study can help researchers using interdisciplinary models to communicate about the validity of models, the validation, and their assessment.

We hope that our map will support social scientists to get more involved in CAS, after all socio-technical and socio-ecological systems refer to social agents and processes which are generally not the expertise of, say, engineers and ecologists. While we focus specifically on CAS models, we expect much of Fig. 2 to be generalizable to interdisciplinary modelling applications in general. We think Fig. 2 also useful for modelling within the social sciences, in particular in modelling social systems where individual agents are nested in (sub)groups and societies, and inputs from multiple social sciences (psychology, anthropology and sociology) are needed. In addition, a better understanding of CAS modelling can help the social sciences to better understand human–environment interactions, e.g. how behaviours like physical distancing may be influenced by lay-out and COVID-19 warnings in public spaces.

The first weakness of the present paper lies in the stereotyping necessary for general statements and in the selection of textbooks. The textbooks are by no means representative of all research methodology and modelling textbooks, but they do reflect heterogeneity in both focus and audience. We investigated both ABM and system dynamics modelling, because both techniques can help understand the behaviour of system’s components but we did not investigate big data and machine learning techniques. The inductive reasoning enabled by these is insufficient to describe and explain how the components of the system operate. As interdisciplinary research aims to understand socio-technical and socio-ecological components and interactions, it cannot rely on inductive reasoning, but needs transparent models to build and test theory. Furthermore, without describing the interactions within the complex system, it is difficult to assess the generalizability of the model which hampers the utility of black-box models for informing policy. We presented one illustration (Libre et al, 2015) that modelled a specific real-world system. Many models are more abstract. For example, the Schelling model (Schelling 1971) suggests a mechanism that might cause segregation, but does not model a specific real-world case. Some of the steps in Fig. 2 may not apply to abstract models, since these rely less on real-world data as input.

We noticed that producing knowledge during the modelling process, through verification, validation or sensitivity analysis, may be seen as research through designing as we know it from the interdisciplinary field of landscape architecture (Lenzholzer et al. 2017). Indeed, research through designing does justice to the creative component of modelling in interdisciplinary research. The potential of this analogy needs further investigation. In the present paper we have omitted reliability, precision, responsiveness and accuracy. All these concepts relate to measurement properties, either of the instruments used to generate model inputs, the results of the modelling or the properties of the model as a product. Another opportunity for future research would be to incorporate these concepts in the presented map.

Concluding, this paper provided a framework that linked validity to validation in the context of interdisciplinary CAS modelling. The proposed terminology allows to describe how interdisciplinary modelling research relies on the validity of primary research used for input, how the quality of the modelling depends on structural and behavioural validation, and, how the assessment of the validity of the model is informed by these types of validation plus research with independent data. We hope that the framework will help to assess and improve the validity, and consequently, utility of models for interdisciplinary CAS research, and for informing policy advice related to CAS.

## Appendix 1

See Appendix Table

**Table 3:** Summary of which keywords appeared in which textbooks

B#	B1	B2	B3	B4	B5	B6	B7	B8	B9	B10	B11
Authenticity	−	+	−	−	−	+	+	−	−	−	−
Concept validity	−	−	−	−	−	−	+	−	−	−	−
Construct validity	+	+	+	−	+	+	+	−	−	−	−
Content validity	+	−	−	−	+	+	+	−	−	−	−
Concurrent validity	+	+	−	−	+	−	+	−	−	−	−
Convergent validity	+	+	−	−	−	−	−	−	−	−	−
Confirmability	−	+	−	−	−	−	+	−	−	−	−
Credibility	−	+	−	−	+	+	+	−	−	−	−
Criterion validity	+	−	−	−	+	+	−	−	−	−	−
Dependability	−	+	−	−	−	−	+	−	−	−	−
Discriminant validity	+	−	−	−	−	−	−	−	−	−	−
Ecological validity	+	+	−	−	+	−	−	−	−	−	−
External validity	+	+	+	+	+	+	−	−	−	−	−
Face validity	+	+	−	−	−	+	+	−	−	−	−
Generalizability	−	+	−	−	+	+	−	−	−	−	−
Instrument validity	−	−	−	−	+	−	+	−	−	−	−
Internal validity	+	+	−	+	+	+	−	−	−	−	−
Measurement validity	+	+	−	−	−	−	+	−	−	−	−
Predictive validity	+	+	−	−	+	+	+	−	−	−	−
Responsiveness to change	+	−	−	−	−	−	−	−	−	−	−
Sensitivity	+	−	−	−	−	−	−	−	−	−	−
Sensitivity analysis	+	−	−	−	−	−	−	+	+	+	−
Specificity	+	−	−	−	−	−	−	−	−	−	−
Statistical validity	−	−	+	−	−	+	−	−	−	−	−
Transferability	−	+	−	−	+	−	+	−	−	−	−
Trustworthiness	−	+	−	−	−	+	+	−	−	−	−
Behavioural validation	−	−	−	−	−	−	−	−	−	+	+
Structural validation	−	−	−	−	−	−	−	+	−	−	+
Correlational models	−	−	−	−	−	−	−	−	−	−	+
Causal-descriptive models	−	−	−	−	−	−	−	−	−	−	+
Boundary adequacy	−	−	−	−	−	−	−	−	−	+	+
Dimensional consistency	−	−	−	−	−	−	−	−	−	+	+
Direct-extreme condition testing	−	−	−	−	−	−	−	−	+	+	+
Parameter confirmation	−	−	−	−	−	−	−	−	−	+	+
Structure confirmation test	−	−	−	−	−	−	−	−	−	+	+
Comparing model and empirical data	−	−	−	−	−	−	−	−	+	−	−
Validation of abstract models	−	−	−	−	−	−	−	−	+	−	−
Validation of facsimile models	−	−	−	−	−	−	−	−	+	−	−
validation of middle-range models	−	−	−	−	−	−	−	−	+	−	−
Macro-level regularities criterion of	−	−	−	−	−	−	−	−	+	−	−
Verification	−	−	−	−	−	−	−	+	+	−	−
Robustness analysis	−	−	−	−	−	−	−	+	−	−	−
Uncertainty analysis	−	−	−	−	−	−	−	+	−	−	−

A plus sign indicates the keyword was present, a minus sign indicates it was not. The B1–B11 refer to the textbooks numbered 1–11 in Table 1

^3^.
